# Inter-Observer Variation in Delineating the Pharyngeal Constrictor Muscle as Organ at Risk in Radiotherapy for Head and Neck Cancer

**DOI:** 10.3389/fonc.2021.644767

**Published:** 2021-03-09

**Authors:** Imran Petkar, Dualta McQuaid, Alex Dunlop, Justine Tyler, Emma Hall, Chris Nutting

**Affiliations:** ^1^ Head and Neck Unit, Royal Marsden NHS Foundation Trust, London, United Kingdom; ^2^ Division of Radiotherapy and Imaging, Institute of Cancer Research, London, United Kingdom; ^3^ Joint Department of Physics, The Institute of Cancer Research and The Royal Marsden NHS Foundation Trust, London, United Kingdom; ^4^ Department of Physics, Royal Marsden NHS Foundation Trust, London, United Kingdom; ^5^ Clinical Trials and Statistics Unit, Institute of Cancer Research, London, United Kingdom

**Keywords:** head and neck cancer, pharyngeal constrictor muscles, dysphagia, dysphagia-optimized IMRT, normal tissue complication probability, inter-observer variation, oropharyngeal cancer, DARS

## Abstract

**Background and Purpose:**

To evaluate the inter-observer variation (IOV) in pharyngeal constrictor muscle (PCM) contouring, and resultant impact on dosimetry and estimated toxicity, as part of the pre-trial radiotherapy trial quality assurance (RTQA) within DARS, a multicenter phase III randomized controlled trial investigating the functional benefits of dysphagia-optimized intensity-modulated radiotherapy (Do-IMRT) in pharyngeal cancers.

**Methods and Materials:**

Outlining accuracy of 15 clinicians’ superior and middle PCM (SMPCM) and inferior PCM (IPCM) were retrospectively assessed against gold standards (GS) using volume, location, and conformity indices (CIs) on a pre-trial benchmark case of oropharyngeal cancer. The influence of delineation variability on dose delivered to the constrictor muscles with Do-IMRT and resultant normal tissue complication probability (NTCP) for physician-scored radiation-associated dysphagia at 6 months was evaluated.

**Results:**

For GS, SMPCM, and IPCM volumes were 13.51 and 1.67 cm^3^; corresponding clinician mean volumes were 12.18 cm^3^ (SD 3.0) and 2.40 cm^3^ (SD 0.9) respectively. High IOV in SMPCM and IPCM delineation was observed by the low DICE similarity coefficient value, along with high geographical miss index and discordance index values. Delineation variability did not significantly affect the mean dose delivered to the constrictors, relative to the GS plan. Mean clinician NTCP was 24.6% (SD 0.6), compared to the GS-NTCP of 24.7%.

**Conclusions:**

Results from this benchmark case demonstrate that inaccurate PCM delineation existed, even with protocol guidelines. This did not impact on delivered dose to this structure with Do-IMRT, or on estimated swallowing toxicity, in this single benchmark case.

## Introduction

Irradiation of the pharyngeal constrictor muscle (PCM) is implicated with post-radiotherapy (RT) dysphagia in head and neck cancer (HNC), resulting in increased risks of aspiration, prolonged feeding tube dependency, and worsened health-related quality of life (1, 2). Sparing RT dose to this critical dysphagia/aspiration at risk structure (DARS) is paramount to improve long-term swallowing function. The successful implementation of swallow-sparing RT techniques in HNC is therefore reliant on contouring accuracy of this critical swallowing organ at risk (SW-OAR) to facilitate optimal avoidance during RT planning. DARS (CRUK/14/014) is a phase III randomized controlled trial in the UK that is currently investigating the functional benefits of reducing dose to the constrictors with dysphagia-optimized intensity-modulated RT (Do-IMRT), relative to standard IMRT, in cancers of the oropharynx and hypopharynx (3). Heterogeneity in PCM definition among clinicians within the study may lead to erroneous interpretation of RT-related morbidity, and consequently affect the assessment and interpretation of the primary endpoint of the study. In addition, variable contouring may lead to inaccurate correlation between PCM dose-volume parameters and radiation-associated morbidity, and any subsequent parameters generated for predicting swallowing toxicity may be misleading (4, 5).

As part of the RT quality assurance (RTQA) program for DARS, clinicians were expected to successfully complete a pre-trial contouring case before enrolling patients in the study at their centers. Our aims in this study were to analyze the differences in PCM delineation between head and neck oncologists within the context of this pre-trial contouring program, evaluate the dosimetric impact of inter-observer variability (IOV) with Do-IMRT, and lastly, to determine the clinical impact of outlining variability on estimated swallowing toxicity.

## Materials and Methods

### DARS Pre-Trial Contouring RTQA Program

The pre-trial quality exercise included a contouring test case with T2N2c base of tongue tumor (AJCC 7^th^ edition), in which clinicians from 15 centers were required to delineate the clinical target volumes (CTV) and OARs, including superior and middle PCM (SMPCM) as one structure and inferior PCM (IPCM) as a separate structure. Do-IMRT planning was not required on the pre-trial contouring test case; a separate pre-trial planning test case with pre-outlined CTVs and OARs was supplied to participating centers, who were expected to submit a protocol-compliant Do-IMRT plan. The DARS trial RT protocol document described in detail the RTQA process for outlining and planning to facilitate the delivery of high-quality RT within the study. In particular, there was a comprehensive section on PCM delineation, which was based on the guidelines by Christianen et al. (6), and the slice-by-slice contouring atlas produced by the PATHOS RTQA team (7). Centers downloaded the planning computed tomography (CT) scan dataset, with gross tumor volume pre-outlined, in digital imaging and communications in medicine—RT from the RTQA website. All completed cases were reviewed by the DARS RTQA team. Each submission was visually evaluated by the chief investigator to determine whether it conformed to the requirements of the trial protocol, and were classified as “per protocol,” “acceptable variation with comments for future cases,” or “unacceptable variation.” Individualized feedback, as per the “Global Harmonization Group” guidelines (8), was subsequently provided to each clinician along with either an approval or a request for resubmission of contours. Participating centers were only permitted to recruit patients after successful completion of the pre-trial QA exercises.

### Contour Analysis

This study was a retrospective quantitative and qualitative analysis of variation in PCM delineation from the initial submission of 15 clinicians, relative to a gold standard (GS) PCM contour, in order to evaluate the IOV that would have existed for this novel structure if a pre-trial quality assurance program did not exist. Re-submitted contours were not evaluated in this study and will form part of another study. The GS in this study was created by a senior radiation oncologist who was part of the panel of international experts that developed and published the consensus guidelines for CT-based delineation of OARs, including the PCM, in HNC. The completed test case outlines were exported to the research version of RayStation treatment planning system (version 5.9.9, RaySearch Medical Laboratories, AB Stockholm, Sweden) for analysis within this study. IOV was assessed using whole volume assessment, surface-based mean and maximum distance to agreement (DTA) (9), and volume-based conformity indices (CIs). These metrics were written in python programming language and implemented in RayStation as a script that could be executed for each study dataset. The following CIs were retrospectively evaluated to determine the concordance between clinician and GS contours (**Supplementary Figure 1**):

- Dice similarity coefficient (DSC): reflects the overall agreement between the volumes of two contours. An ideal score is 1, indicating perfect overlap with the GS contour (10) (9). A score of > 0.7 is considered to represent good agreement between two contours (11–13).
- Geographical miss index (GMI): indicates the amount of GS contour not included in the clinician contour. An ideal score is 0, implying no “under-contouring” (14).
- Discordance Index (DI): indicates the amount of clinician outlining not included in the GS contour. An ideal score is 0, indicating no “over-contouring” (15).

Contouring variation for the brainstem and parotid glands, 2 routinely delineated OARs in HNC, were also determined to serve as a useful comparator for the constrictors.

In addition to whole-volume conformity analysis described above, a slice-by-slice CIs evaluation of clinician PCM (slice DSC (s-DSC), s-GMI etc.) was additionally carried out (**Supplementary Figure 2**) to identify volume variation on a slice-by-slice basis of the constrictor muscle delineation (14), using the equation described in **Supplementary Figure 1**. Positional variation on each slice was additionally established by evaluating the maximum distance from the surface of GS delineation to the clinician contour in the anterior, posterior, right lateral, and left lateral direction on each slice.

These metrics were not used as tools to provide feedback for submissions within the real-time pre-trial RTQA and were solely used for the purpose of this study.

### Dosimetric Analysis

**Figure 1:**
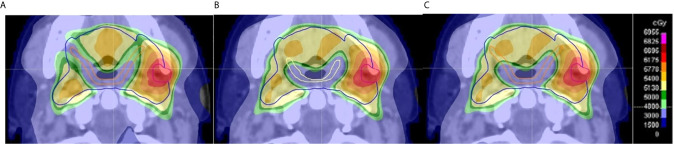
Example of evaluation of dose delivered to pharyngeal constrictor muscle (PCM) based on clinician contours on an axial CT slice. **(A)** The GS Do-IMRT plan based upon the gold standard (GS) superior and middle PCM (SMPCM) (orange) and GS IPCM contour was created to record the dose-volume histogram (DVH) for this SW-OAR; **(B)** shows a clinician Do-IMRT plan that was generated using the clinician’s SMPCM (yellow) and IPCM delineation to derive the relevant dose metrics; **(C)** GS SMPCM and GS IPCM contour was superimposed on the clinician’s Do-IMRT plan in **(B)** to allow their DVHs to be derived. This was then compared to the original DVH obtained in **(A)**. The presence of variation between the GS and clinician contour, as in this slice, would highlight differences in dose delivered. In this example, it can be seen that there was less sparing of GS SMPCM laterally on clinician Do-IMRT plan compared to GS plan.

Centers were not expected to generate Do-IMRT plans for the pre-trial contouring test case. A three-step methodology was therefore adopted to quantify the dosimetric impact of IOV in PCM contouring for the test case, as shown in **Figure 1**. In step 1, GS mean dose to the constrictors was determined by generating a GS Do-IMRT plan using GS target volumes and OARs including SMPCM and IPCM. This was the reference plan against which clinician plans were compared. In step 2, 15 clinician Do-IMRT plans based on individual clinician’s delineation of the constrictor muscle were created in order to determine corresponding mean doses. For these plans, GS target volumes and non-swallowing OARs were used for RT optimization, rather than clinician volume delineation. This step facilitated the evaluation of possible dosimetric impact that could be attributed only due to differences in PCM definition by the 15 oncologists. In step 3, GS-SMPCM and GS-IPCM structure sets were superimposed on clinician RT plans constructed in step 2, and the mean dose delivered to the GS contours on these plans was derived. This step allows the evaluation of whether the dose to the PCM on RT plans created using clinicians’ definition of the constrictor muscle represents what the GS delineation receives. Measuring this outcome is relevant to study, as it is possible that the reported dose to this critical swallowing OAR may not be a true reflection of dose received in the presence of contouring errors, and therefore subsequently reported toxicity outcomes may be inaccurate.

The Do-IMRT planning technique of DARS for oropharyngeal tumors has been previously described elsewhere (3). In brief, the technique aims to spare dose to the constrictors by setting a mandatory mean dose of < 50 Gy to the volume of SMPCM (PlanSMPCM), together with an optimal constraint of < 20 Gy to the volume of IPCM (PlanIPCM) lying outside the high dose clinical target volume. A dose of 65.1 Gy in 30 fractions over 6 weeks was to be delivered to the therapeutic planning target volume (PTV1), and 54 Gy in as many fractions to the prophylactic PTV2.

The GS and clinician RT plans were generated with volumetric-arc therapy, consisting of two 360°arcs with mirrored collimator angles of 30° and 330° respectively, and optimized using the collapse cone v3.4 algorithm in RayStation. The planning objectives and optimization process used for each clinician plan was similar to that used for the reference GS plan.

### Predicted Swallowing Toxicity Analysis

The normal tissue complication probability (NTCP) for physician-scored RTOG > grade 2 radiation-associated dysphagia at 6 months with Do-IMRT was determined by applying the predictive model of Christianen et al. (16–18), in which mean dose to the superior PCM and supraglottic larynx were predictors of toxicity. Following on from the methodology used to determine the dosimetric impact of IOV in contouring, three swallowing toxicity models were accordingly calculated—GS-NTCP, based on GS Do-IMRT plan; clinician NTCP based on their plans; and lastly the estimated risk of dysphagia when the reference GS contours were superimposed on the investigator RT plans.

### Statistical Analysis

Analysis was performed using Statistical Package for the Social Sciences (SPSS) version 25. Variables with normal distribution were reported as mean and 95% confidence interval (95% CI), while those not normally distributed were reported as median and interquartile range (IQR). One sample t-tests were calculated for GS dosimetry and estimated toxicity to assess for clinician variation.

## Results

GS-SMPCM and GS-IPCM volumes were 13.5 and 1.7 cm^3^ respectively. Clinicians’ mean SMPCM and IPCM volumes were 12.2 cm^3^ (95% CI 10.5–13.8, standard deviation (SD) 3.0, range 8.5 to 13.2) and 2.4 cm^3^ (95% CI 1.9–2.9, SD 0.9, range 1.3 to 4.4) respectively. Mean volumes for clinicians’ ipsilateral parotid, contralateral parotid, and brainstem were 32.5 cm^3^ (95% CI 30.7–34.4, SD 2.2; GS 35.2 cm^3^), 37.1 cm^3^ (95% CI 35.4–38.9, SD 2.3; GS 37.1 cm^3^), and 22.6 cm^3^ (95% CI 19.9–25.3, SD 3.5; GS 25.7 cm^3^) respectively.

**Figure 2:**
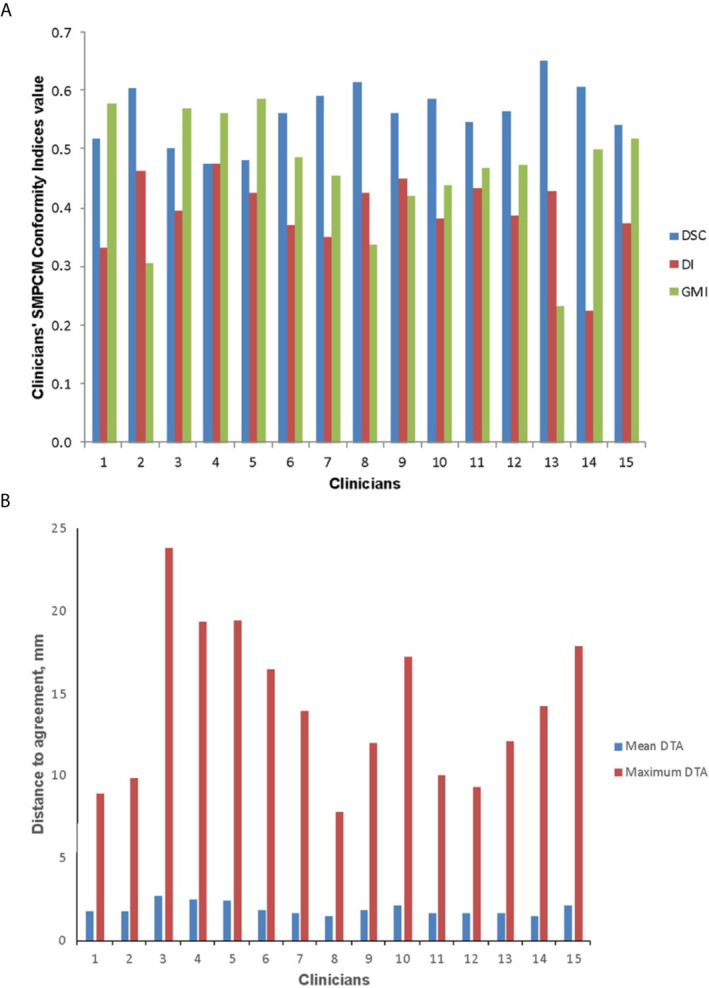
Conformity indices **(A)** and distance to agreement (DTA) **(B)** results for clinicians**’** superior and middle pharyngeal constrictor muscle (SMPCM) contours DI, discordance index; DSC, DICE similarity co-efficient; GMI, geographical miss index.

Low DSC, high GMI, and DI values were observed for clinicians’ SMPCM and IPCM contours (**Table 1**). 2 of the 15 clinicians achieved a DSC > 0.70 for their IPCM delineation (**Supplementary Figure 3**), and none for SMPCM contouring (**Figure 2**). The GMI values indicated that a mean of 6.3 cm^3^ (range 3.2–8.0 cm^3^) and 0.5 cm^3^ (range 0.2–0.9 cm^3^) of the GS–SMPCM and –IPCM contours were outside the clinicians’ outlining respectively. In other words, on average 46.6 and 30.0% of GS–SMPCM and –IPCM volumes were not included in the clinicians’ delineation. The DI values, particularly for IPCM, imply substantial over-contouring. For 11 (73%) SMPCM and 3 (20%) IPCM contours, the maximum DTA was > 1 cm relative to the corresponding GS contour. In comparison, there was good agreement for the non-swallowing OARs, with DSC of > 0.80 for both parotids and BS (**Table 2**).

**Table 1:** Values for different conformity indices for superior and middle pharyngeal constrictor muscle (SMPCM) and inferior pharyngeal constrictor muscle (IPCM).

Structure	SMPCM					IPCM				
	DSC	DI	GMI	Mean DTA (mm)	Max DTA (mm)	DSC	DI	GMI	Mean DTA (mm)	Max DTA (mm)
Range	0.48–0.65	0.23–0.48	0.23–0.59	1.5–2.8	7.8–23.8	0.31–0.72	0.31–0.78	0.14–0.54	0.9–5.0	3.6–15.8
Median	–	–	–	1.8	–	–	–	–	1.4	5.3
IQR	–	–	–	1.7–2.2	–	–	–	–	1.2–2.6	4.5–9.8
Mean	0.56	0.40	0.46	–	14.2	0.57	0.49	0.33	–	–
95% CI	0.53–0.59	0.36–0.43	0.40–0.52	–	11.5–16.8	0.51–0.63	0.41–0.56	0.26–0.40	–	–
SD	0.05	0.06	0.10	0.04	0.47	0.11	0.13	0.13	0.11	0.37

DI, discordance index; DSC, DICE similarity co-efficient; DTA, distance to agreement; IQR, interquartile range; GMI, geographical miss index; SD, standard deviation; 95% CI, 95% confidence interval.

**Table 2:** Values for different conformity indices for ipsilateral and contralateral parotid gland, and brainstem.

Structure	Ipsilateral parotid gland					Contralateral parotid gland				
	DSC	DI	GMI	Mean DTA (mm)	Max DTA (mm)	DSC	DI	GMI	Mean DTA (mm)	Max DTA (mm)
Range	0.85–0.89	0.05–0.15	0.12–0.2	1.3–2.1	7.2–15.5	0.82–0.90	0.08–0.17	0.08–0.20	1.1–1.6	6.0–12.2
Median	0.87	–	–	–	–	–	–	0.10	–	–
IQR	0.87–0.87	–	–	–	–	–	–	0.10-0.13	–	–
Mean	–	0.09	0.16	1.6	1.18	0.87	0.11	–	1.4	9.3
										
95% CI	–	0.07–0.12	0.14–0.19	1.4–1.8	9.1–14.5	0.85–0.89	0.09–0.14	–	1.2–1.6	7.8–11.1
SD	0.01	0.03	0.03	0.02	0.33	0.02	0.03	0.04	0.02	0.2
**Structure**	**Brainstem**									
	**DSC**		**DI**		**GMI**		**Mean DTA (mm)**		**Max DTA (mm)**	
Range	0.74–0.88		0.05–0.25		0.15–0.38		1.2–3.3		4.1–11.9	
Mean	0.82		0.12		0.23		2.0		0.73	
95% CI	0.78–0.86		0.06–0.17		0.17–0.29		1.5–2.6		5.5–9.1	
SD	0.05		0.07		0.08		0.07		0.23	

DI, discordance index; DSC, DICE similarity co-efficient; DTA, distance to agreement; IQR, interquartile range; GMI, geographical miss index; SD, standard deviation; 95% CI, 95% confidence interval.

**Figure 3:**
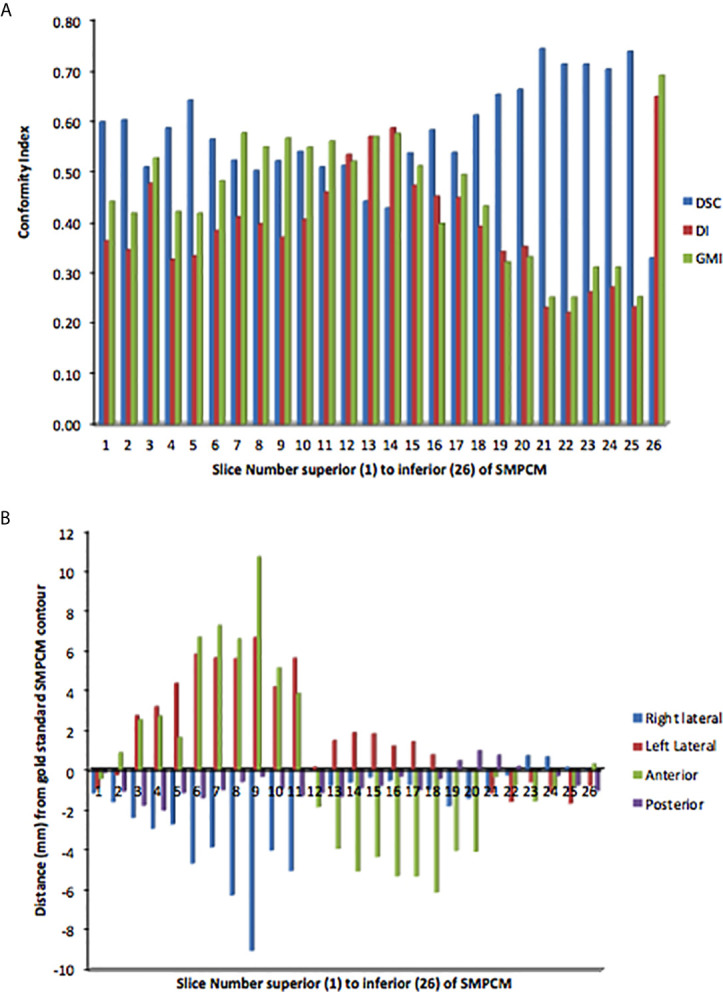
Slice-by-slice conformity **(A)** and positional **(B)** analysis of clinicians’ superior and middle constrictor muscle (PI-SMPCM) contours DI, discordance index; DSC, DICE similarity co-efficient; GMI, geographical miss index.

For clinicians’ SMPCM, the median s-DSC was 0.57 (IQR 0.51–0.65); s-GMI, 0.46 (IQR 0.33–0.55); and s-DI 0.39 (IQR 0.33–0.46) (**Figure 3**). Corresponding values for IPCM were 0.70 (IQR 0.50–0.76); 0.22 (IQR 0.16–0.46); and 0.34 (IQR 0.23–0.59) respectively (**Supplementary Figure 4**). There was considerable variation in defining the superior-inferior extents of both SMPCM and IPCM relative to GS, with perfect concordance observed in only one IPCM and three SMPCM delineations respectively. Apart from the caudal-most slice, the highest agreement with the GS-SMPCM contours was observed inferiorly for slices 21–25, with median s-DSC > 0.7 and low values of s-GMI (0.25) and s-DI (0.23) respectively. Positional analysis for SMPCM showed that the largest variation was noted mid-way between the superior and inferior slices in the lateral directions predominantly.

GS and all clinician Do-IMRT plans achieved the mandatory target volume and OAR dose constraints. GS doses to the PTV1 (median), PTV2 (median), brainstem (maximum dose), contralateral parotid (mean dose), ipsilateral parotid, and spinal cord (maximum dose) were 65.3 Gy, 54.4 Gy, 40.7 Gy, 31.5 Gy, 32.8 Gy, and 37.2 Gy respectively. Corresponding means of the clinician doses on clinician Do-IMRT plans were 65.4 Gy, 54.5 Gy, 41.1 Gy (95% CI 39.9–42.2), 31.3 Gy (95% CI 31.1–31.4), 33.1 Gy (95% CI 32.9–33.3), and 40.0 (95% CI 39.1–40.9) respectively.

**Figure 4:**
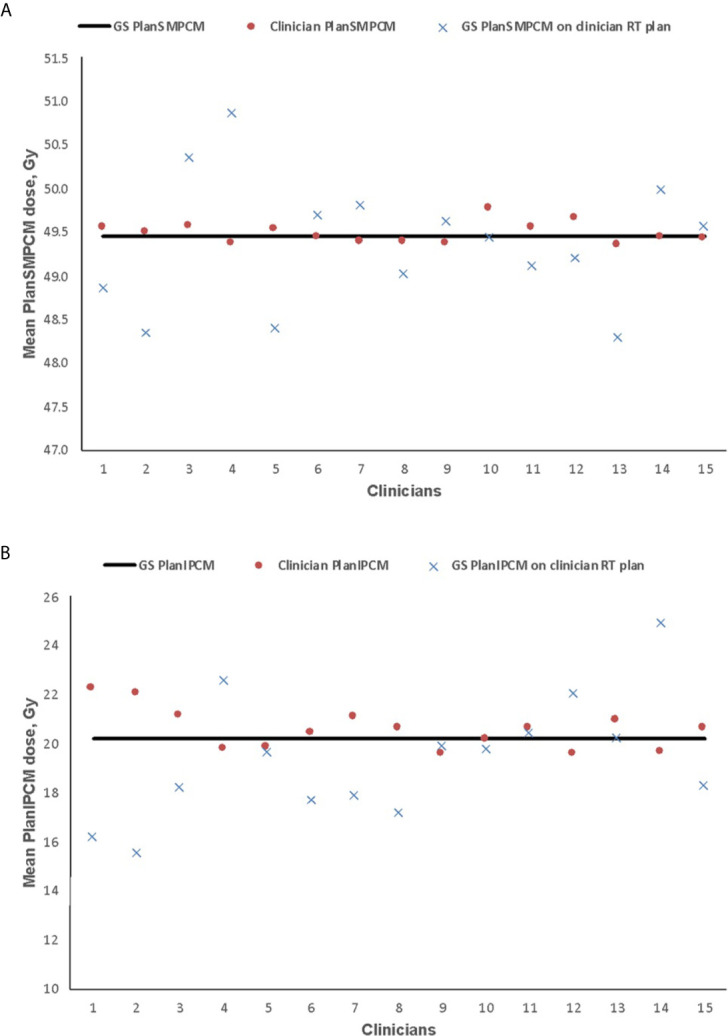
Mean dose delivered to plan superior and middle pharyngeal constrictor muscle (PlanSMPCM, **A**) and inferior PCM (PlanIPCM, **B**) with clinician dysphagia-optimized intensity modulated therapy (Do-IMRT) plans, and the gold standard (GS) contour superimposed on the clinicians’ Do-IMRT plan The horizontal line represents the mean dose delivered to the structures on the GS plan, based on GS contours.

GS PlanSMPCM dose was 49.5 Gy. There was no difference between this reference dose and average of the mean dose to clinician PlanSMPCM on clinician Do-IMRT plans (49.5 Gy, 95% CI 49.4–49.6, SD 0.1; p = 0.7). The mean dose to the GS PlanSMPCM when the GS constrictor contours were superimposed on clinician plans was, on average, 0.1 Gy lower than the GS dosimetry and not statistically significant (49.4 Gy, 95% CI 49.0–49.8, SD 0.7; p = 0.5). For 3 clinician PlanSMPCM contours, the dose delivered to GS delineation on clinician Do-IMRT plans was found to be greater than the mandatory Do-IMRT dose constraint of < 50 Gy (**Figure 4**). The mean of the clinician mean PlanIPCM dose was 20.6 Gy (95% CI 20.1–21.0, SD 0.8), and was not statistically inferior to the GS dose of 20.2 Gy (p = 0.1); corresponding value for GS contour superimposed on clinician plan was 19.4 Gy (95% CI 18.0–20.8, SD 2.4; p = 0.2).

**Figure 5:**
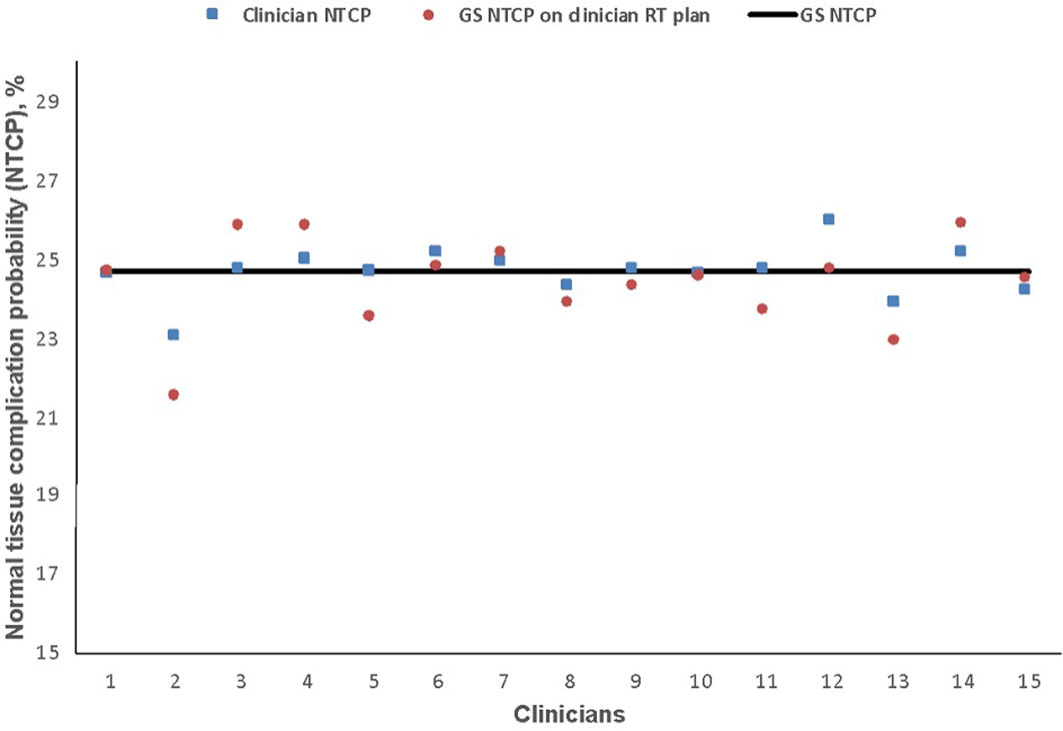
Normal tissue complication probability (NTCP) values for physician-scored radiation-associated dysphagia at 6 months based on clinicians’ dysphagia-optimized intensity-modulated radiotherapy (Do-IMRT) plans. The horizontal line represents the NTCP value for the gold standard (GS) Do-IMRT plan, based on GS pharyngeal constrictor muscle contours.

The estimated risk of dysphagia is shown in **Figure 5**. GS-NTCP was 24.7%. The difference between GS and clinician mean NTCP was 0.1% (95% CI 24.3–25.0, SD 0.6; p= 0.7); corresponding difference between the GS-NTCP and when the GS contour was superimposed on clinician plans was 0.3% (95% CI 23.7–25.0, SD 1.1; p= 0.3).

## Discussion

To our knowledge, this is the first study to explore variation in PCM delineation, and its impact on predicted swallowing toxicity, in the UK. We have shown that clinicians’ conformity to the GS volume for both SMPCM and IPCM was poor with the first submission, as evidenced by the variable whole volumes where there was 1.5-fold and 3.4 fold-difference between clinicians’ volumes respectively, low DSC and high DI and GMI scores. Whole-volume CIs, however, do not provide sufficient information about differences in size, shape, or location that may exist between 2 volumes. Similar CIs values for different contours, therefore, do not necessarily indicate that the contours are identical. For instance, one clinician achieved a DSC of 0.65 (ranked 1^st^ of 15), GMI of 0.23 (ranked 1^st^ of 15), but a DI of 0.43 (ranked 11^th^ of 15) for SMPCM delineation. Visual assessment of the contours, however, showed that the delineation did not extend laterally to encompass the pterygoid muscle as specified in the trial protocol. On the other hand, no protocol violation was identified for another clinician who scored a DSC of 0.62 (2^nd^ of 15), GMI of 0.34 (3^rd^ of 15), and DI of 0.43 (10^th^ of 15) for SMPCM delineation. Outlining errors for the constrictor muscles may therefore be missed if whole-volume CIs alone were used to establish levels of agreement between contours. The addition of slice CIs provides a quantitative, and more objective, evaluation by facilitating the identification of slices of disparity between clinician and gold standard, which might lead to more robust analysis. The s-CIs values for clinician IPCM delineation observed in this study imply that the relatively poor corresponding whole volume CIs values were largely due to uncertainty in defining the superior and inferior extent of this structure.

Our study also showed that systematic delineation errors occurred despite the presence of a detailed contouring protocol and delineation atlas. For instance, three clinicians wrongly assumed the caudal edge of cricoid cartilage as the inferior border of the IPCM. Spatial assessment for SMPCM delineation additionally demonstrated that concordance with the GS contour was poor in the middle section of this structure, where the lower s-GMI and s-DSC compared to the mean overall GMI and DSC suggested under-outlining as the contouring error. Visual assessment of the discordant slices identified that under-outlining was often due to failure to extend the delineation of SMPCM laterally to encompass the pterygoid muscle.

Certain factors may have influenced the poor PCM CIs values, relative to GS. In contrast to the brainstem and parotids where CT provides sufficient soft tissue contrast for delineation, the PCM is not readily visualized on CT and its contouring is therefore reliant on accurate interpretation of guidelines based on different anatomical landmarks, which is likely to have contributed to the higher degree of variation observed in this study. For instance, the cranial and caudal extent of PCM was subject to substantial IOV implying uncertainty in identifying the tip of the pterygoid plates and the lower edge of the arytenoid cartilages, which may be due to unfamiliarity with identifying these on CT. It is also pertinent to consider the relatively smaller volume of the constrictors relative to the standard OARs when interpreting the differential CIs values. CIs are more sensitive to the smaller volumes, as a few missing or extra voxels on one contour is sufficient to skew their values. On the other hand, they are more forgiving for larger volumes such as the parotids where a relatively larger variation is required to demonstrate a comparable CIs result.

There are only a few studies that have investigated PCM contouring variability. Feng *et al*. found significant IOV among three clinicians in fractional overlap (intersection volume divided by union volume) for PCM (mean 0.5), when the muscle was delineated on three separate occasions (19). Alterio *et al*. additionally showed that there was increased intra- and inter-observer variability in delineation of the superior pharyngeal constrictor muscle, along with lower adherence compared to the corresponding MRI-contoured muscle, among 34 HN oncologists (20); the study group did not assess the dosimetric impact of IOV. It is difficult to make comparisons with the above studies, due to differences in the respective methodologies and delineation guidelines. Our work has not only identified that IOV for contouring of PCM existed, similar to the published literature, but also established the areas of maximum variation from the reference contour within the study population. The described measurements of IOV in this study were not used during the DARS pre-trial RTQA, where feedback to the clinicians was based on visual evaluation of their submissions by the quality assurance team. Implementing such measurements in addition may lead to targeted analysis of areas of high discordance, and facilitate the introduction of semi-automated assessment measures (15).

The PCM often falls in the region of high dose and steep dose gradients. Inaccuracy in the contouring of this swallowing OAR could potentially under-report the mean dose received if the voxels are erroneously placed outside of the high dose region, or have the converse effect if extra voxels are incorrectly placed in the high-dose regions. We therefore studied two surrogate clinical outcome measures, namely differences in dosimetry and estimated risk of swallowing toxicity at 6 months, to determine the impact of any contouring variation in the constrictor muscle on subsequent toxicity burden, relative to the reference contour. Despite establishing volumetric, overlap, and spatial variability in contouring of the PCM, we found that there was minimal impact on the mean dose delivered to this structure with Do-IMRT and risk of persistent swallowing dysfunction compared to GS. Such an outcome would suggest that variability in the delineation of this swallowing OAR does not impact on the dose delivered with Do-IMRT, which would be consistent with results of Feng et al. and that pre-trial contouring QA for this structure may not be necessary (21). Before drawing firm conclusions to that effect, it is pertinent to consider certain limitations in this study. This analysis was conducted on a single benchmark case with minimal target volume-PCM overlap, and it is possible that the clinical outcomes with PCM contouring variability could differ with increasing number of cases and/or greater overlap. Furthermore, the ball diameter used to contour the PCM with certain clinicians was wider than the 3 mm used for the GS contour; at the time of DARS pre-trial exercise, there was no agreed consensus about the width of this muscle for the purpose of delineation. Consequently, there was a larger dose gradient on their plans relative to the GS plan, explaining why the mean doses to the GS on some plans was smaller. Variability in supraglottic larynx delineation was not assessed in this study and it remains possible that outlining uncertainties for this structure may lead to different toxicity outcomes than the one presented in this study. Finally, the NTCP model applied in this study was not validated for the RT treatment technique used here.

In this study, an “expert-defined” gold standard was used as the benchmark contour, against which all contours were compared. Therefore, there may be an element of bias introduced into our results. Currently, there remains no consensus regarding definition of a gold standard volume within the context of pre-trial quality assessment, with published studies choosing between GS contour such as in this study, or a mathematically derived consensus contour. Similarly, there could be a debate about the reproducibility of our GS Do-IMRT plan; however the same would hold true for the clinician Do-IMRT plans too. The intent of this study was to examine the IOV and subsequent dosimetric and clinical impact, and we feel the possibility of OAR and plan variability would always remain irrespective of the chosen reference structure and plan. We did not analyze the differences in dose delivered to the constrictors with standard IMRT and Do-IMRT for each clinician outlining. This was not the aim of this study, and therefore the potential impact of delineation variability on dose delivered to the two arms of DARS trial, and consequent implications on trial results, cannot be determined.

In conclusion, qualitative and quantitative assessments demonstrated considerable IOV in the delineation of the PCM on a single pre-trial benchmark case, due to a combination of inaccurate interpretation of the contouring protocol and unfamiliarity with radiological landmarks. The inconsistent definition of PCM did not have a detrimental impact on dosimetry or estimated toxicity, but it is premature to make such a conclusive assumption on a single test case alone. Future work would involve analysis of contouring from standard and Do-IMRT plans of treated trial patients and associations with clinical toxicity outcomes.

## Data Availability Statement

The original contributions presented in the study are included in the article/**Supplementary Material**. Further inquiries can be directed to the corresponding author.

## Author Contributions

IP—first author and corresponding author for this manuscript. Original idea for this research, data collection, and analysis, wrote the first draft and final version of this manuscript. DM—data collection, revised draft manuscript and approved final version of the manuscript. AD—data collection, revised draft manuscript and approved final version of the manuscript. JT—data collection, revised draft manuscript and approved final version of the manuscript. EH—data analysis, revised draft manuscript and approved final version of the manuscript. CN—senior author, data analysis, revised draft manuscript and approved final version of the manuscript. All authors contributed to the article and approved the submitted version.

## Conflict of Interest

CN is the chief investigator of DARS trial.

The remaining authors declare that the research was conducted in the absence of any commercial or financial relationships that could be construed as a potential conflict of interest.
