# Supplementary figures and images for: Inter-Observer Variation in Delineating the Pharyngeal Constrictor Muscle as Organ at Risk in Radiotherapy for Head and Neck Cancer
Source: Front Oncol. 2021 Mar 9;11:644767. doi: 10.3389/fonc.2021.644767 (PMC7985335; doi:10.3389/fonc.2021.644767)

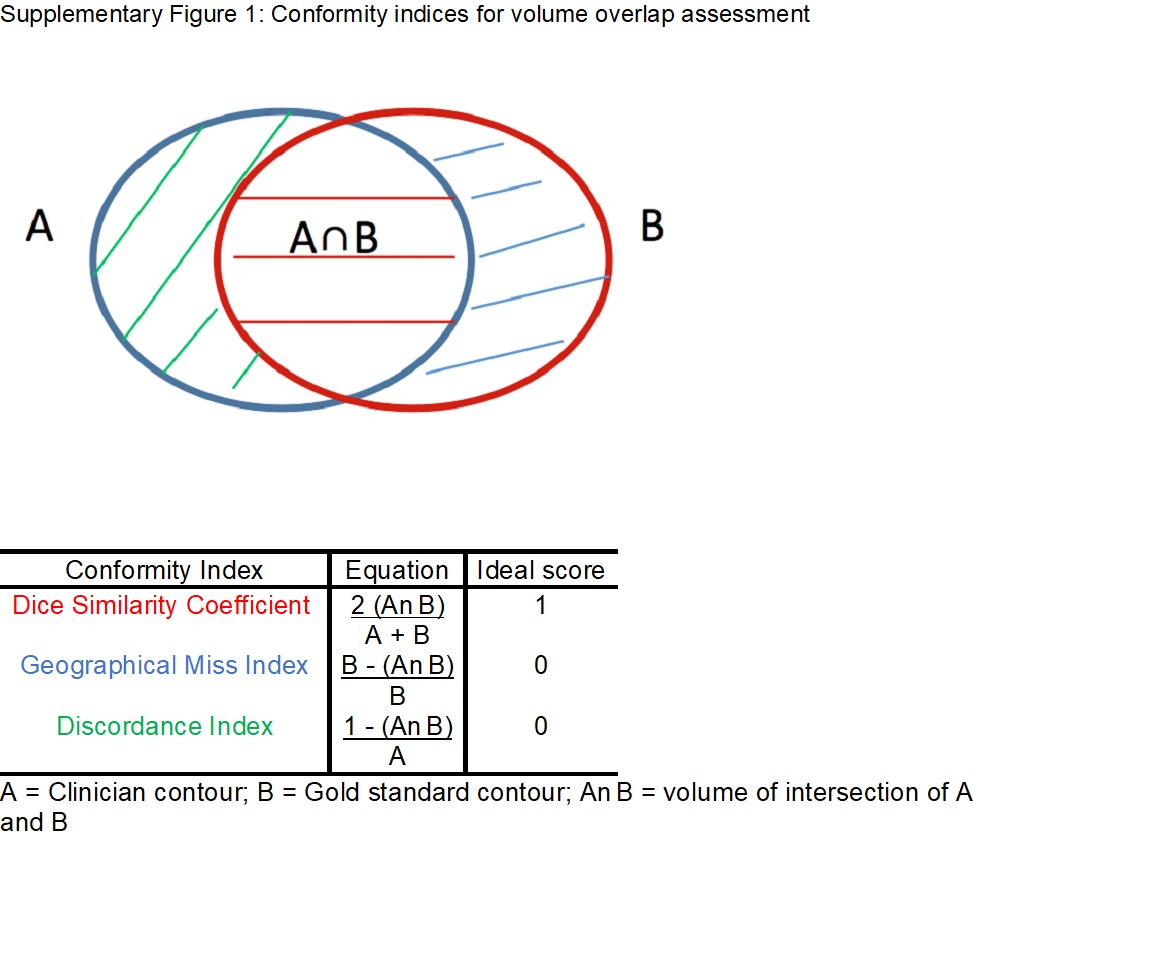

Supplement: Supplementary file 1 [file Image_1.jpeg]

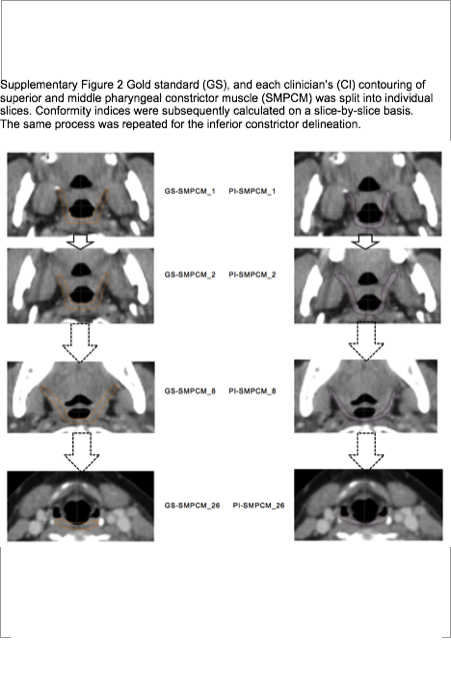

Supplement: Supplementary file 2 [file Image_2.jpeg]

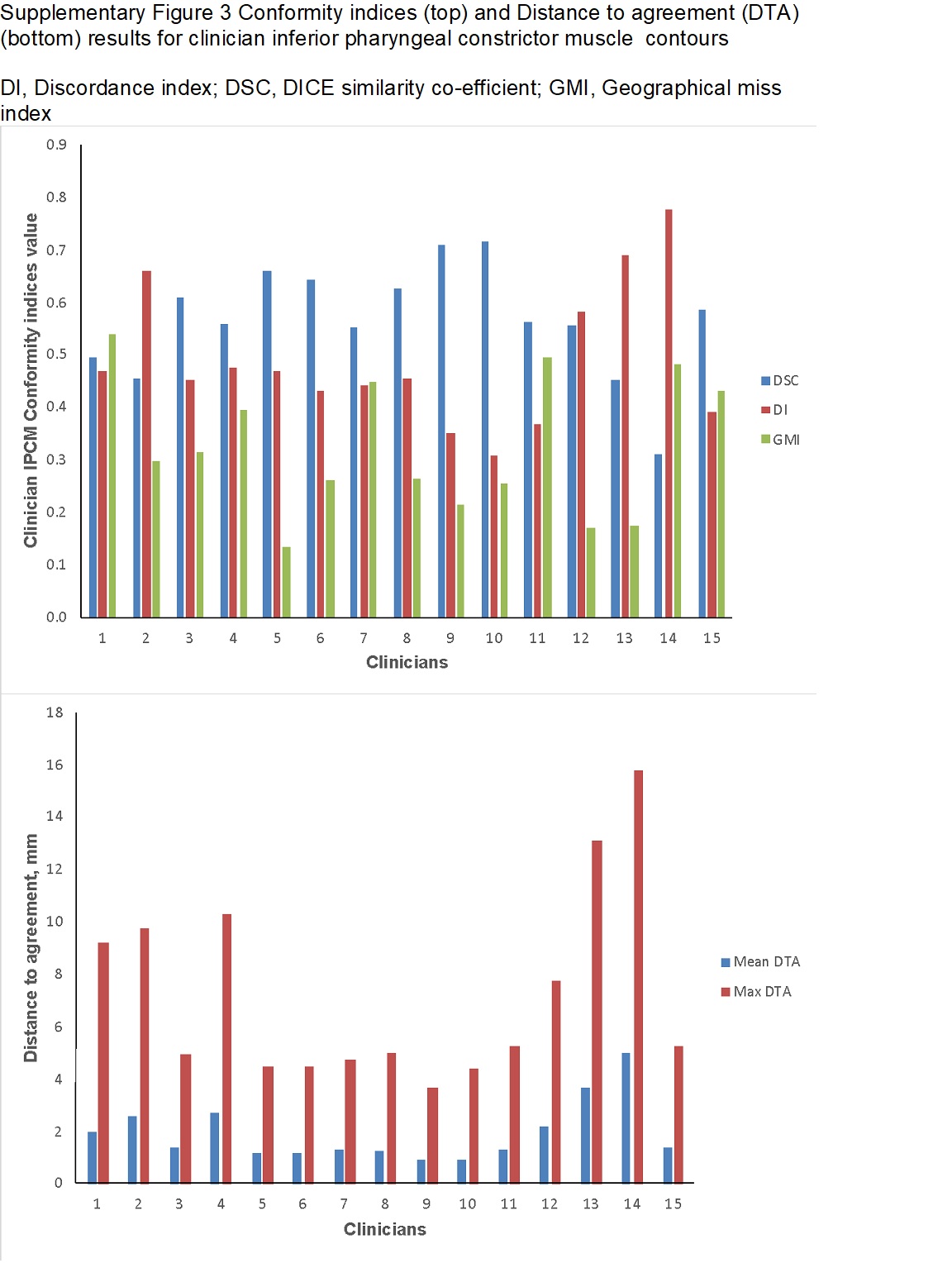

Supplement: Supplementary file 3 [file Image_3.jpeg]

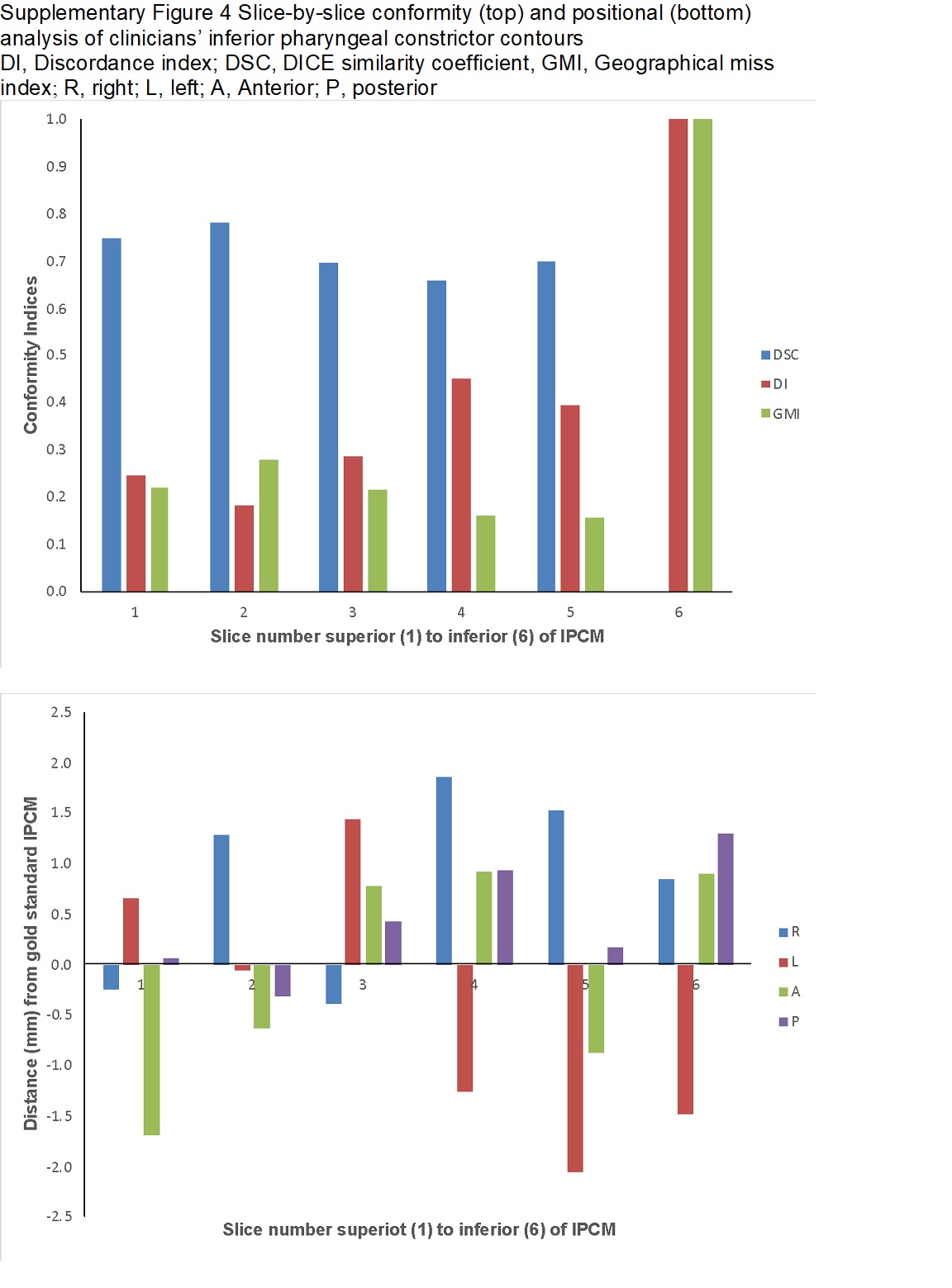

Supplement: Supplementary file 4 [file Image_4.jpeg]
